# Multimodal generative AI for pre-practicum mental health support competency development: a human-supervised framework for education–childcare partnerships

**DOI:** 10.3389/fpsyt.2026.1922251

**Published:** 2026-08-31

**Authors:** Wenlan Xie, Chunye Xiang, Fan Wu, Xingda Chen

**Affiliations:** 1 Department of Research Affairs, Ningbo Childhood Education College, Ningbo, China; 2 School of Teacher Education, Ningbo University, Ningbo, China; 3 Heqin Preschool Education Institute, Ningbo Childhood Education College, Ningbo, China

**Keywords:** education-childcare partnerships, generative artificial intelligence, implementation science, mental health support competence, multimodal interaction, pre-practicum training, pre-service childcare professionals, responsive caregiving

## Abstract

Generative artificial intelligence (GenAI) and multimodal interaction are attracting growing attention in mental health-related education and care. At the same time, pre-service childcare professionals are increasingly expected to observe and respond sensitively to infants’ and toddlers’ emotional signals, attachment needs, separation reactions, and interaction patterns. These contextual and ethically sensitive capacities require more than lectures or procedural training. Authentic practicum remains indispensable, but safeguarding, consent, privacy, placement availability, supervisory capacity, and the unpredictability of naturally occurring situations can limit safe rehearsal. Drawing on research on large language models, conversational agents, responsive caregiving, competency-based assessment, work-integrated learning, implementation science, and health AI governance, this conceptual analysis proposes five design domains for education–childcare partnerships: competency mapping, multimodal simulation, AI-supported reflective feedback, practicum readiness assessment, and partnership governance. Multimodal GenAI is positioned as human-supervised learning infrastructure that may extend established simulation through adaptive scenario variation, contingent interaction, multimodal cue integration, and scalable formative feedback. The proposed architecture includes a developmental-fidelity gate under which synthetic infant- or toddler-like materials would be reviewed against predefined anatomical, affective, temporal, cultural, and pedagogical criteria before use. The framework defines non-clinical mental health support competence through five observable dimensions and specifies human authority, data-minimization boundaries, and a staged research pathway beginning with a candidate Pre-Practicum Mental Health Support Competency Assessment (PP-MHSCA) blueprint. The proposed competency levels, readiness categories, critical-error rules, scoring procedures, and thresholds are candidate specifications requiring stakeholder review, pilot testing, standard setting, and empirical validation before implementation. Comparative studies with conventional simulation and lecture/case preparation, followed by prospective tests of practicum performance, can determine whether the approach adds educational value. The framework offers a cautious and governable bridge to practicum without displacing educators, mentors, or real-world experience. The framework is grounded in broadly applicable professional standards and governance principles, with China proposed as the initial context for implementation and empirical evaluation. Evidence generated through this process is expected to inform the framework’s subsequent adaptation, transfer, and application in other national and regional contexts.

## 1 Introduction

Early childhood care and education is an important setting for supporting young children’s psychological well-being, although it is not a psychiatric service. Emotional regulation, attachment-related behavior, separation responses, social engagement, and help-seeking signals are often observed during routine care. The nurturing care framework identifies responsive caregiving, safety, health, nutrition, and early learning as interconnected foundations of development (1, 2). Accordingly, pre-service childcare professionals need more than procedural skills: their role includes creating emotionally safe environments, observing development and learning in context, communicating respectfully with families, documenting evidence, and exercising professional responsibility (3).

These relational and situational competencies cannot be developed through lectures alone. Students need repeated opportunities to observe ambiguous cases, rehearse communication and decision-making, receive feedback, and reflect before assuming responsibility in real settings. Reliance on authentic practicum for repeated training may expose children and families to unnecessary observation, data collection, premature interpretation, or poorly prepared responses. A protected pre-practicum environment is therefore needed in which mistakes can support learning without directly affecting children.

Multimodal GenAI offers a cautious way to extend established simulation before practicum. Under educator supervision, synthetic, staged, or de-identified cases can support scenario variation, family or mentor conversations, reflective questioning, and draft feedback while institutions test validity, privacy, bias, misinformation, and automation risks (4–6). The relevant question is not whether AI can produce realistic material, but whether it adds educational value under defined limits. This paper therefore positions GenAI as a human-supervised learning infrastructure rather than a system for child monitoring, diagnosis, institutional evaluation, or autonomous readiness decisions, consistent with responsible behavioral-health AI and WHO guidance (4, 7, 8). This boundary is reinforced by emotion research showing that facial movements are not universally diagnostic of specific emotions, that visual context influences facial-emotion recognition, and that children’s interpretation of facial expressions develops gradually rather than mapping directly onto discrete emotion categories (9–11).

The framework links five design domains with intended learning, bounded AI functions, retained human authority, and shared responsibility for data, feedback, remediation, and practicum transition. Implementation evaluation considers acceptability, adoption, appropriateness, feasibility, fidelity, cost, reach, and sustainability; equity, workload, and unintended consequences are treated as additional framework-specific outcomes (12–14). The main contributions are a definition of non-clinical mental health support competence, a cautious entry point for GenAI, and a falsifiable research agenda using credible comparators and prospective validity testing.

## 2 Scope and conceptual positioning

This section defines the target professional group and mental health support competence, explains why it should be developed before practicum, and clarifies the potential role of multimodal GenAI.

### 2.1 Target professional group

In this framework, the term pre-service childcare professionals refers to students or other learners in vocational or higher-education pathways preparing for supervised practice in center-based childcare services, including vocational, associate-level, and undergraduate programs where applicable. The primary group is learners preparing to work with infants and toddlers, particularly children under 3 years, in childcare institutions or comparable settings. It may also include career entrants or learners with experience in adjacent early childhood education, caregiving, family-service, or related settings, provided that they have not assumed independent responsibility in the target childcare role and are completing the required entry-to-practice preparation. Thus, pre-service describes their status in the target role rather than the absence of previous employment or related experience.

These learners undertake preparation and placement under qualified educators and designated on-site childcare mentors or supervisors. Their permitted responsibilities include supervised responsive caregiving, developmentally informed observation, routine strengths-based communication with families and colleagues, objective documentation, and timely escalation through established procedures. They may participate in safeguarding, consultation, and referral processes only under supervision.

### 2.2 Mental health support competence

Mental health support competence comprises the knowledge, skills, dispositions, and professional judgment used to create emotionally safe environments, observe and respond to distress, communicate with families, document concerns, and seek qualified guidance or referral. It is demonstrated through decisions and actions rather than self-report alone. Crying, withdrawal, aggression, silence, sleep difficulty, or separation distress may reflect development, temperament, health, family transitions, culture, relationships, or the care environment; no isolated behavior is diagnostically decisive. Competent practice considers persistence, severity, developmental level, functional impact, context, and caregiver–child relationships, while describing rather than labeling and consulting when concerns persist (15).

The framework distinguishes four related but non-equivalent activities. Responsive caregiving addresses the child’s immediate emotional and regulatory needs; developmental observation records observable behavior, context, duration, frequency, intensity, functional impact, and change from the child’s usual pattern; safeguarding activates established procedures when there are indicators of possible harm, abuse, neglect, or immediate risk; and mental-health assessment involves clinical interpretation, diagnosis, and treatment planning by appropriately qualified professionals. The proposed educational scope covers responsive caregiving and developmental observation, together with supervised safeguarding escalation and consultation; it does not authorize trainees to conduct clinical assessment or make autonomous diagnoses or referrals.

### 2.3 Why the pre-practicum phase matters

Practicum is indispensable but may pose risks when students enter without sufficient preparation. Weak preparation can lead to escalating responses, stigmatizing family communication, incomplete documentation, poor safeguarding judgment, blurred role boundaries, or excessive reliance on unstructured mentor feedback. Work-integrated learning also shows unequal participation and outcomes across student groups and variable access to placement opportunities (16). A shared pre-practicum baseline therefore serves both safety and equity by reducing dependence on the variable opportunities of individual sites.

Pre-practicum preparation is best understood as a developmental bridge rather than an administrative orientation. Students can establish a common vocabulary, rehearse sensitive interactions, practice documentation and escalation, and learn to use supervision through structured, repeatable activities before working with children and families.

### 2.4 Why multimodal GenAI?

Childcare judgment is inherently multimodal: words, vocal tone, posture, facial expression, interaction patterns, routines, environment, family context, and institutional rules may all matter. Text cases capture only part of this complexity. Established simulation already uses multiple media; the potential addition of multimodal GenAI is the adaptive and scalable orchestration of scenario variants, contingent dialogue, and individualized prompts within educator-defined objectives.

These affordances create additional risks. More realistic media can be mistaken for developmentally valid representations, and audiovisual materials increase privacy and biometric sensitivity. Modality use is justified only when it contributes information required by a defined competency. Synthetic materials reduce reliance on identifiable recordings but may still reproduce bias, stereotypes, or developmental error; as constructed learning materials, their validity requires evidence rather than assumptions. Section 6 specifies the corresponding data, review, transparency, and student-rights requirements.

Developmental fidelity cannot be inferred from photorealism. Infant pose research shows that adult-derived models may fail because infant proportions and movement differ, while virtual-infant and digital-child projects illustrate the need for domain-specific, multidisciplinary development and validation (17–20). Synthetic infant- or toddler-like materials therefore require separate review of anatomy, movement, affect, vocalization, timing, relational contingency, cultural plausibility, and pedagogical usefulness. In the absence of a unified benchmark, pilot validation can use expert-approved reference materials, prespecified age- and cue-specific criteria, inter-rater agreement, and developmental-error severity. Materials that fail the criteria are withheld, revised, restricted, or retired.

### 2.5 Primary context of application and international transferability

The framework is grounded in broadly applicable professional standards and governance principles and is proposed for initial implementation and empirical evaluation in the Chinese context, with a focus on the target professional group defined in Section 2.1. This initial application is intended to examine its feasibility, contextual appropriateness, and implementation conditions, while accumulating practice-based evidence to inform further development.

International professional standards and related frameworks are used in this study primarily as resources for conceptual development and comparative analysis, rather than as substitutes for national laws and regulations, professional standards, or institutional requirements. Implementation in China should therefore be contextually aligned with relevant national childcare standards and guidelines (21–23), the Personal Information Protection Law and the Law on the Protection of Minors (24, 25), mandatory reporting requirements (26), applicable practicum and practice-based training requirements (27), as well as relevant provincial and institutional regulations. Implementation experience and empirical evidence generated in the Chinese context may subsequently provide a reference for adapting the framework to other countries and regions.

## 3 Theoretical and empirical evidence

### 3.1 Conceptual-analysis methodology

This study employed conceptual analysis to clarify the meaning, dimensions, boundaries, and educational implications of pre-practicum mental health support competence.

The analysis was informed by Petrina’s conceptual-analysis framework (28), including the identification of a conceptual problem, clarification of semantic variation, mapping of conceptual dimensions, definition of scope and boundaries, and development of an integrated conceptual representation. The central problem addressed in this study was the fragmentation of relevant competencies across different fields, without a coherent framework for pre-practicum preparation.

The analysis drew on five literature streams: GenAI and large language models in mental health; multimodal simulation and conversational practice; responsive caregiving and early childhood mental health; competency-based education and work-integrated learning; and AI governance and implementation science. Relevant evidence was also drawn from teacher-education simulation (29), GenAI-supported virtual- and simulated-patient methodologies (30–32), and infant and early childhood mental health competency and consultation frameworks (33–36).

Sources were selected purposively according to their theoretical relevance, conceptual contribution, contextual applicability, and ability to clarify professional boundaries. The extracted concepts were compared, differentiated, and mapped onto five design domains. Overlapping elements were integrated, whereas elements outside the non-clinical and pre-practicum scope were excluded or treated as boundary conditions. Further details of the search strategy, source selection, concept extraction, author roles, and synthesis process are provided in **Supplementary Appendix S1**, which, for analytical transparency, also classifies sources into four evidence categories—direct component, adjacent, analogical, and normative—according to their proximity to the target context and the function they served, and identifies the references in each category along with the limits on transfer. Accordingly, the outputs of the analysis should be interpreted at three levels: evidence-informed foundational principles; a conceptually derived five-domain architecture; and conceptually derived operational specifications that require further stakeholder review and empirical validation.

### 3.2 GenAI in mental health: capabilities, risks, and role boundaries

Reviews of LLMs in mental health report expanding capabilities alongside unresolved problems of validity, safety, bias, generalizability, and deployment readiness (4, 5, 37–39). In childcare education, GenAI may support educator-designed preventive and relational learning by generating and adapting scenarios, organizing cues for supervised reflection, rehearsing communication, comparing alternative responses, and assisting with planning and documentation. Multimodal scenarios may integrate linguistic, vocal, visual, gestural, contextual, and relational cues. However, current systems remain vulnerable to hallucination, bias, and contextual misunderstanding (4, 5, 37–39), while early childhood literature raises additional concerns about developmental alignment, child-centred implementation, and the limited evidence base in this setting (40, 41). Accordingly, multimodal cues are suitable only for supervised observation and professional discussion. In particular, facial movements should not be treated as context-free readouts of discrete emotion, and limited behavioral signals should not be converted automatically into clinical labels (9–11, 40, 41).

### 3.3 Multimodal GenAI-supported simulation and conversational practice

Conversational agents can support sustained, interactive exchanges (6), but therapeutic findings cannot be transferred directly to childcare education. Here, conversational GenAI is a simulation modality for practicing family communication, mentor consultation, descriptive observation, documentation, and routine de-escalation—not a therapeutic agent. The rationale derives from established simulation: structured practice, repetition, and feedback can improve learning while allowing errors to be examined without exposing children or families (29, 42).

Conventional role-play, video cases, standardized encounters, and scripted simulations remain credible alternatives. GenAI may add open-ended dialogue, rapid variation, and individualized prompts while preserving educator-defined objectives. Early virtual-patient studies suggest feasibility and short-term learning or usability benefits, but the evidence is heterogeneous, mainly outside childcare, and limited longitudinally (30–32). It supports comparative testing, not claims of superiority. Conventional approaches may be preferable when stability, reproducibility, expert control, or subtle embodied interaction is central. GenAI is most defensible for supervised formative rehearsal when its adaptive functions address a defined learning problem and outputs can be challenged and revalidated.

#### 3.3.1 Incremental educational value of GenAI

GenAI is not required for the core educational functions of simulation, reflection, feedback, competency mapping, or readiness assessment. These functions can be delivered through educator-facilitated role-play, standardized cases, peer discussion, or conventional video-based simulation. GenAI is educationally relevant only where it addresses identifiable limitations of these lower-risk alternatives.

Its potential incremental value lies primarily in four areas. First, GenAI may support the rapid generation and adaptation of scenarios that vary in child age, family context, communication style, emotional intensity, safeguarding relevance, and ambiguity. Second, it may enable repeated practice with dynamically evolving interactions rather than fixed scripts or single-response cases. Third, it may allow learners to compare multiple plausible response pathways and examine how different wording, timing, or escalation decisions alter the course of an interaction. Fourth, it may provide timely prompts for structured reflection between educator-supervised sessions, thereby extending practice opportunities without replacing professional feedback.

These affordances do not justify GenAI use by themselves. GenAI-supported activities should be introduced only when they demonstrate educational benefit beyond lower-risk alternatives and when those benefits outweigh additional concerns relating to accuracy, privacy, bias, developmental fidelity, cost, and educator workload. Where equivalent outcomes can be achieved through role-play, standardized cases, conventional simulation, or direct educator feedback, the lower-risk approach should be preferred.

### 3.4 Responsive caregiving and the content of mental health support competence

Responsive caregiving provides the substantive content of the framework. It emphasizes sensitive, timely, reciprocal, developmentally appropriate support within everyday routines (1, 2). Competence involves noticing verbal and nonverbal signals, considering multiple contextual explanations, responding without escalation, adapting to individual needs, and communicating with families and professionals. The proposed construct therefore comprises five observable dimensions: emotionally safe relationships; contextual observation and recognition of distress; proportionate responses; sensitive family and interprofessional communication; and professional judgment in documentation, safeguarding, boundaries, and help-seeking or referral. These dimensions draw on NAEYC, ZERO TO THREE, and infant and early childhood mental health competency frameworks (3, 33–36). Reflective and culturally responsive judgment cuts across all five dimensions (3, 33–36); critical AI literacy is additionally required when AI-supported tools are used (43). Because meaning varies with age, development, culture, health, family circumstances, setting, and relational history, AI-identified features can only prompt supervised reasoning; they cannot determine child mental health, caregiving quality, or student competence. The construct excludes mental-health screening or diagnosis, treatment, autonomous risk or referral decisions, inference of hidden mental states from isolated cues, and independent case management.

### 3.5 Competency development, assessment, and a testable pre-practicum pathway

Competency-based assessment focuses on demonstrated performance, and graded models can represent progression beyond binary competent/not-yet-competent judgments (44). Mental health support competence cannot be inferred from knowledge, confidence, satisfaction, or activity completion alone. It is demonstrated through observation, contextual interpretation, proportionate support, sensitive communication, responsible documentation, role boundaries, and timely help-seeking. For conceptual and developmental purposes, the framework proposes a provisional four-level model—unsafe, emerging, competent, and advanced—as a candidate representation of progression. The developmental principle is evidence-informed, whereas the number of levels, their descriptors, and the boundaries between them require stakeholder review, rater testing, and empirical examination. Within this candidate model, unsafe performance includes confidentiality or safeguarding failures, stigma, unsupported diagnosis, or action beyond role boundaries; advanced performance integrates evidence, uncertainty, alternatives, and revision after feedback. Assessment examines both action and the reasoning, ethics, contextual sensitivity, and reflection underlying it.

#### 3.5.1 Proposed pre-practicum mental health support competency indicator framework

An evidence-informed crosswalk of NAEYC, ZERO TO THREE, and infant and early childhood mental health competency documents (3, 33–36) informed the provisional organization of the five dimensions. Its purpose is to organize the non-clinical capacities that pre-service childcare professionals may need before practicum to observe, respond, communicate, document, and escalate concerns safely within their professional role. **Table 1** translates them into concise observable indicators and assessment approaches, showing that competence must be sampled across interaction, observation, communication, documentation, and safety-critical judgment. CLASS Infant/Toddler and Q-CCIIT/QCIT may offer complementary evidence about interaction quality, but they do not cover the full construct (45–47). **Table 1** is therefore intended as a framework for instrument development and empirical testing, not a validated scale.

**Table 1:** Core indicators and assessment approaches for pre-practicum mental health support competence.

Competency dimension	Core observable indicators	Main assessment methods
1. Emotionally safe environments and responsive relationships	Warm and consistent responses; co-regulation; support for emotional expression and belonging; preventive environmental design.	Simulation stations; structured observation; situational-judgment test (SJT); reflective rationale.
2. Contextual observation and recognition of distress	Multi-source and multi-context observation; distinction between transient and persistent or severe patterns; objective documentation; recognition of uncertainty and red flags. Descriptive observation is separated from diagnostic inference.	Knowledge test and SJT; multimodal observation; documentation exercise; reflective rationale.
3. Proportionate response to distress	Developmentally appropriate, non-escalating support; adaptation of timing and strategy; judgment about when to observe, intervene, seek supervision, or request additional support.	Simulation stations; role-play; SJT; observation checklist; reflective rationale.
4. Sensitive family and interprofessional communication	Strengths-based, non-stigmatizing, and culturally responsive language; factual sharing of observations; invitation of family perspectives; collaboration with mentors and relevant professionals.	Simulated family conversations; written communication samples; role-play; reflective rationale.
5. Professional judgment, documentation, safeguarding, and referral	Objective and confidential records; clear role boundaries; recognition of safeguarding and referral thresholds; timely consultation and escalation; ethical and culturally responsive professional judgment. Clinical mental-health assessment and diagnosis remain outside the trainee role.	Documentation task; safeguarding and referral simulations; safety-critical checklist; SJT; reflective rationale.

In GenAI-supported training, critical AI literacy is treated as a cross-cutting enabling capability and is explicitly taught and assessed across the five dimensions (43, 48). Assessment can require students to recognize hallucinated or fabricated content, verify feedback against observation and professional standards, resist automation bias, identify concealed uncertainty or role overreach, refuse to enter identifiable child or family data into unapproved systems, and explain AI limitations to mentors or families. These behaviors can be examined through situational-judgment items, deliberately flawed feedback, and reflective rationales.

#### 3.5.2 Candidate PP-MHSCA assessment blueprint

Building on the proposed Pre-Practicum Mental Health Support Competency Indicator Framework, the PP-MHSCA is presented as a candidate assessment blueprint for future instrument development, not as a validated instrument or a current basis for practicum-readiness decisions. A future pilot version could integrate a brief knowledge and situational-judgment test, four to six standardized simulation stations, reflective rationales, and a safety-critical checklist. Candidate stations could address separation distress, difficulties with regulation, family communication, safeguarding, documentation, and consultation or referral. These station topics are illustrative rather than a finalized station set; their content, modality, duration, prompting format, scoring indicators, assessor requirements, and sampling plan would be developed iteratively with stakeholders and tested empirically.

Any future pilot administration would require scoring by at least two trained human raters, with blinding used wherever feasible, and provisional four-level behavioral anchors corresponding to the five dimensions in **Table 1**. Dimensional profiles, critical-error rules, and readiness thresholds would remain developmental until reliability, validity, accessibility, and fairness evidence supported their use. Artificial intelligence may support formative feedback but must not make high-stakes decisions.

#### 3.5.3 Instrument development and validation

A validation program would include competency crosswalks, expert content-validity ratings, cognitive interviews, accessibility and scenario review, pilot item analysis, and transparent standard setting. Reliability evidence covers inter-rater agreement and generalizability across scenarios. Validity evidence includes known-groups comparisons, associations with mentor or supervisor ratings, and complementary relations with CLASS Infant/Toddler or Q-CCIIT/QCIT observations for interaction quality only (45–47). Until sufficient measurement evidence has been established, the PP-MHSCA should continue to be regarded as a candidate assessment blueprint. Inadequate reliability, weak external associations, or systematic bias related to language, disability, culture, or accessibility should trigger revision.

#### 3.5.4 Link to the empirical testing pathway

Following satisfactory construct development and measurement validation, PP-MHSCA scores may serve as a primary outcome in the empirical testing pathway described in Section 4.7. Comparative effectiveness studies would proceed only after adequate evidence of scoring reliability, validity, feasibility, and equity has been established. These studies could examine whether GenAI-supported preparation improves validated competency outcomes, retention, and generalization to novel cases relative to prespecified comparators.

Predictive validity would be evaluated in a subsequent prospective phase by examining whether pre-practicum PP-MHSCA scores are associated with independently assessed practicum performance. Evidence supporting the broader pathway would therefore require both satisfactory measurement properties and demonstrable educational value. Measurement failure would prompt revision of the construct, assessment tasks, scoring system, or readiness thresholds, whereas null or inconsistent effectiveness findings would prompt reconsideration of the intervention or its implementation conditions.

Future evaluation should therefore compare GenAI-supported training not only with lecture-based instruction, but also with credible lower-risk active-learning alternatives, such as educator-led role-play, standardized cases, and conventional video simulation. The relevant question is not whether GenAI-supported learners improve over time, but whether GenAI produces additional educational benefit beyond these established approaches.

Measurable outcomes may include performance in unfamiliar or varied scenarios, transfer of learning across contexts, quality of reflective reasoning, recognition of safeguarding and referral boundaries, response flexibility, feedback timeliness, learner engagement, educator preparation time, scalability, accessibility, equity, and the frequency of unsafe or inappropriate responses. Incremental value should be judged against both educational outcomes and implementation costs and risks.

#### 3.5.5 Equity in competency development and assessment

Equitable opportunities to acquire and demonstrate competence are essential. GenAI simulation can expand access to repeated practice where placements are limited, yet it may also widen disparities in digital access, language, AI literacy, accessibility for students with disabilities, or cultural familiarity. Accessible interfaces, language support, equivalent non-AI task formats, and reasonable participation alternatives help separate the target competence from digital familiarity. Evaluation examines access, completion, ratings, remediation, and appeals across relevant groups, with construct-irrelevant disparities prompting redesign.

### 3.6 AI Governance and implementation within education–childcare partnerships

AI governance and implementation science define the institutional conditions for introducing this pedagogy. WHO and UNICEF guidance emphasize human autonomy, safety, transparency, accountability, equity, and children’s best interests (7, 8, 49). CONSORT-AI and SPIRIT-AI provide complementary requirements for the transparent specification and reporting of AI interventions, human–AI interaction, inputs and outputs, and evaluation procedures (50, 51). At the conceptual level, institutions define educational purpose, permitted functions, human authority, data flows, and workflow integration, while partners allocate responsibility for scenario review, staff preparation, student support, vendors, and incident response.

Implementation science asks whether the intervention can function sustainably in real settings. Proctor outcomes, CFIR, and RE-AIM direct attention to acceptability, adoption, appropriateness, feasibility, fidelity, cost, reach, equity, sustainability, and context (12–14). Technical sophistication has little value if educators distrust outputs, mentors cannot use feedback, students experience surveillance, families object to data practices, or institutions lack time and expertise. Implementation is therefore treated as organizational and pedagogical change involving shared standards, allocated responsibilities, staff preparation, accessible support, and feedback loops through which practice settings revise curriculum, scenarios, assessment, and supervision.

### 3.7 Questions for further research

Existing research has established a substantial foundation in the educational use of generative AI, simulation-based learning, childcare practice, and competency assessment, but integration across these areas remains limited. Further research is particularly needed to clarify how multimodal generative AI can be translated into structured pre-practicum pedagogy, how campus-based simulation can be aligned with authentic childcare expectations and mentor feedback, and how competency assessment can be integrated with equity, governance, and professional boundaries.

Accordingly, this study conceptualizes generative AI as a supervised “boundary infrastructure” linking campus-based preparation with authentic practice and seeks to integrate simulation-based learning, professional standards, human oversight, data protection, equitable learning opportunities, and practicum feedback. Further stakeholder review, contextual implementation, and empirical evaluation are required to examine its feasibility and applicability.

## 4 Proposed framework: five pre-practicum design domains

**Figure 1:**
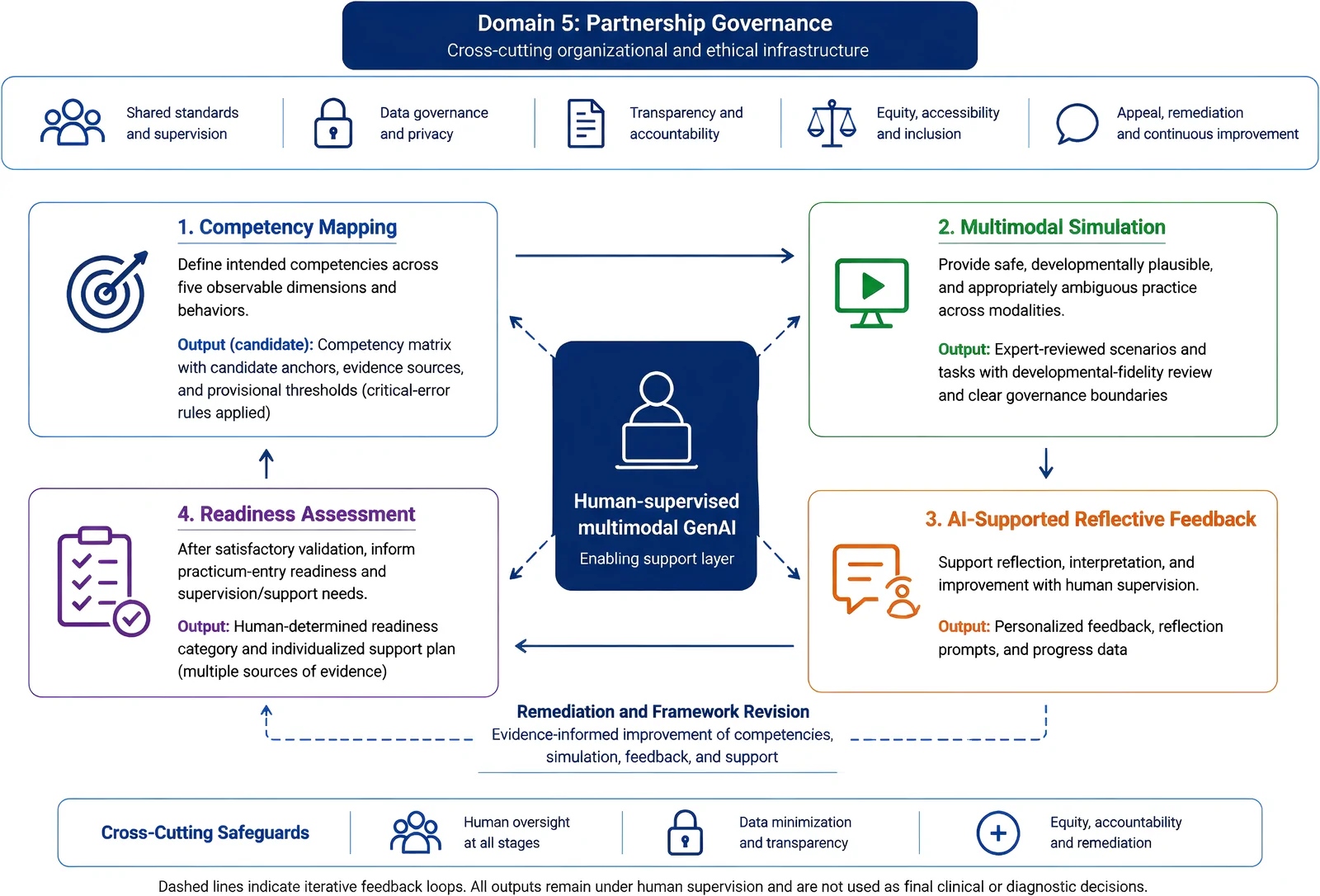
Five-domain pre-practicum framework for multimodal GenAI-supported mental health support competency development. The framework comprises four iterative educational domains—competency mapping, multimodal simulation, AI-supported reflective feedback, and readiness assessment—embedded within a fifth, cross-cutting domain of partnership governance. This configuration is a conceptually derived architecture whose completeness, domain relationships, and contextual applicability require stakeholder examination and empirical evaluation. Evidence generated through readiness assessment informs remediation and the revision of competency expectations, simulation tasks, feedback processes, and individualized supervision or support plans. Human-supervised multimodal GenAI operates as an enabling support layer rather than as an autonomous assessor. Partnership governance provides shared standards and supervision, data governance and privacy, transparency and accountability, equity, accessibility and inclusion, and mechanisms for appeal, remediation, and continuous improvement.

The framework translates this synthesis into five interdependent design domains (**Figure 1**). Competency mapping defines outcomes; simulation provides practice; reflective feedback supports revision; readiness assessment informs entry and supervision; and partnership governance coordinates standards and responsibilities.

The five-domain configuration is the principal conceptually derived output of the analysis. It integrates evidence-informed foundations into a coherent and testable architecture, but its completeness, domain relationships, sequencing, contextual fit, and feasibility require stakeholder examination and empirical evaluation. Readiness evidence feeds back into competency expectations, simulation, feedback, and remediation. Implementation is evaluated through acceptability, appropriateness, feasibility, fidelity, and sustainability (12).

### 4.1 Domain 1: competency mapping

Domain 1 operationalizes the construct specified in Section 3.4 and **Table 1**. Drawing on responsive caregiving, professional standards, infant and early childhood mental health competency documents, assessment principles, and local practicum requirements, educators and practice partners link each dimension to observable behaviors, simulation tasks, feedback criteria, evidence sources, and readiness thresholds. GenAI may help organize or compare draft statements and rubrics, but professionals determine the final definitions and standards.

The principal output of this domain is a competency matrix that connects each dimension with observable behaviors, simulation tasks, feedback criteria, evidence sources, and readiness thresholds. Drawing on general principles of graded competency assessment (44), the present conceptual analysis proposes, for illustrative purposes, the following provisional four-point anchor: 0 = not demonstrated or unacceptable; 1 = emerging and requiring substantial prompting; 2 = competent with minor prompting; and 3 = integrated, context-sensitive, and independently justified. Safety-critical errors are recorded separately and may override aggregate scores under prespecified critical-error rules.

Qualified educators and practice partners establish provisional cut scores through a transparent method, such as a modified Angoff or borderline-group procedure, and recalibrate them against later practicum outcomes. These cut scores and associated thresholds remain candidate decision rules until supported by adequate measurement, fairness, decision-consistency, and consequence evidence. AI may supply draft recommendations, but it does not select or alter readiness thresholds.

### 4.2 Domain 2: multimodal simulation

Once competency expectations have been defined, multimodal simulation provides structured opportunities for students to practice them before working with real children and families. Simulation may include text-based cases, audio role-play, video observation, image-based environmental scanning, branching dialogue, and structured decision-making tasks. Modality selection aligns with the competency being developed rather than with technological availability alone.

GenAI can create controlled variations of cases such as separation distress, withdrawal from peer play, difficult family communication, documentation requests, or possible safeguarding concerns. Variation can expose students to differences in age, context, communication style, culture, environment, and uncertainty without requiring a single predetermined answer.

The purpose of simulation is not to train students to produce a single predetermined answer. It is to develop disciplined observation, sensitive language, contextual reasoning, emotional responsiveness, and appropriate help-seeking habits. Productive scenarios retain some ambiguity because children’s emotional and behavioral signals are rarely self-explanatory. Students identify what is observable, what remains uncertain, what additional information is needed, and when consultation is appropriate.

Simulation materials are classified separately by privacy risk and developmental-fidelity risk. Synthetic text usually presents lower privacy risk, whereas child-like audio, images, animation, or video remain pedagogically sensitive until validated. Real-child materials require documented necessity, lawful authorization, secure access, and qualified review; synthetic materials require evidence that anatomy, movement, affect, vocalization, timing, and context are plausible.

Before instructional use, synthetic infant- or toddler-like media pass a developmental-fidelity gate. The gate defines the age band, cue, and learning purpose; grounds the material in developmental evidence; compares it with an expert-validated reference set; includes multidisciplinary and cultural review; applies prespecified thresholds for content validity, inter-rater agreement, and error severity; and records versions and revalidation. Materials that fail are revised, restricted to error-identification exercises, suspended, or retired. The operational review workflow is specified in Section 4.2.1 and **Table 2**.

**Table 2:** Conceptually grounded provisional developmental-fidelity review protocol for pilot evaluation.

Component	Operational specification	Decision and record
Review team	At minimum, an infant–toddler development specialist and an experienced childcare educator or practicum supervisor review independently; additional safeguarding, cultural or linguistic, accessibility, family, or technical expertise is added as needed.	Reviewer names, roles, conflicts, and independent ratings are recorded; vendor review alone is insufficient.
Evidence package	Age band, learning objective, full scenario or media, developmental sources and reference set, model and version, prompt or settings, human edits, intended and prohibited uses, known limitations, and prior revisions.	An incomplete package is not eligible for approval; all materials are version controlled.
Rating scale	Each dimension is rated 0 = critical or unacceptable; 1 = major revision required; 2 = acceptable with minor correction or restricted supervised use; 3 = acceptable for intended supervised use.	Each rating includes a brief rationale and required correction, if any.
Critical errors	Examples include anatomically impossible or substantially distorted representation; behavior seriously inconsistent with the stated developmental stage or context; unsafe caregiving or safeguarding responses; diagnostic labeling or hidden-state inference from isolated cues; and portrayal of ordinary developmental variation as pathology.	Any rating of 0 results in non-use until the material is corrected and fully re-reviewed.
Acceptance rule	No dimension is rated 0; all core developmental and non-pathologizing dimensions are rated at least 2 by both required reviewers; and all required corrections are completed and documented.	Approval states the permitted use and any restrictions; total or average scores cannot override a critical error.
Disagreement	Reviewers first document and discuss the basis of disagreement. Unresolved disagreement is referred to a third independent qualified reviewer.	Safety, safeguarding, or pathologizing concerns default to non-use until resolved; the final decision and rationale enter the audit record.
Revalidation	Revalidation follows a material change in model, prompt, modality, scenario, age band, language, or cultural context; a fidelity or safety incident; recurring learner misinterpretation; or a predefined periodic review interval (for example, annual review during the pilot phase).	The scope of re-review is proportionate to the change, but critical developmental, non-pathologizing, and safety dimensions are always re-rated.

The developmental, non-pathologizing, safeguarding, and human-review principles are evidence-informed; the rating scale, critical-error definitions, acceptance rules, thresholds, and revalidation intervals are provisional candidate specifications requiring stakeholder and specialist review, pilot testing, and empirical evaluation before routine implementation.

#### 4.2.1 Developmental-fidelity review gate

The gate is a documented human review workflow rather than an automated realism score. For a pilot, review is conducted independently by at least one infant–toddler development specialist and one experienced childcare educator or practicum supervisor. Safeguarding, family, cultural or linguistic, accessibility, and technical expertise are added when relevant; material creators and vendors may provide documentation but cannot be the sole reviewers.

The review package includes the intended age band and learning objective; the complete scenario or media sequence; developmental sources and expert reference materials; model and version; prompt, settings, and human edits; intended and prohibited uses; known limitations; and previous review records. Reviewers examine anatomy and biomechanics, developmental plausibility of behavior, language, vocalization and timing, contextual and relational coherence, non-pathologizing representation, safeguarding and response safety, cultural and accessibility considerations, cross-modal consistency, and pedagogical usefulness.

The rating categories and acceptance rule are provisional governance specifications for pilot evaluation, not validated psychometric thresholds. Their clarity, feasibility, inter-reviewer consistency, and consequences should themselves be evaluated and refined.

### 4.3 Domain 3: AI-supported reflective feedback

Simulation alone does not ensure competence development. Feedback is the mechanism through which simulated performance is interpreted, questioned, and improved. In this domain, GenAI functions primarily as a formative support tool rather than as an assessor with independent authority.

GenAI may summarize a student’s response, compare it with specified rubric criteria, identify omitted safety or family communication considerations, generate reflective questions, suggest alternative wording for sensitive interactions, help mentors draft individualized comments, and organize patterns of progress across repeated simulations. For example, after a student responds to a scenario involving a withdrawn child, the system might ask whether the response distinguished observation from interpretation, considered contextual explanations, avoided diagnostic language, and identified an appropriate point for mentor consultation.

Feedback is tiered by consequence: automated prompts may support low-stakes reflection, whereas supervisor-mediated and readiness-related feedback follows the review, transparency, appeal, and non-punitive-use requirements in Section 6.

Students also evaluate AI feedback itself. When a model labels behavior from a brief scenario, they examine the evidence, missing context, plausible alternatives, and whether the proposed response falls within the childcare role. Critical AI literacy is thus part of professional judgment: recognizing uncertainty, resisting unsupported conclusions, and using digital tools responsibly.

### 4.4 Domain 4: readiness assessment

This domain describes a future use case after satisfactory construct development and measurement validation. During initial development and validation, readiness evidence remains developmental and exploratory and must not determine practicum entry. Once acceptable evidence of reliability, validity, accessibility, and fairness has been established, readiness assessment may integrate evidence from the preceding domains to examine whether a student meets minimum safety and professional requirements and what supervision or further preparation may be needed.

Readiness is not represented by a single AI-generated score. Any future decisions would draw on multiple sources of evidence, including performance across different simulation modalities, reflective writing, structured observation, rubric-based educator review, childcare mentor judgment, student self-assessment, and completion of essential privacy and safeguarding requirements.

For future use after satisfactory validation, **Table 3** presents four provisional human-determined readiness categories derived from dimension-level ratings and critical-error rules.

**Table 3:** Provisional candidate human-determined readiness categories for future practicum-entry research.

Readiness category	Meaning	Illustrative evidence
Not yet ready	A safety-critical threshold is unmet or the evidence is insufficient.	Confidentiality or safeguarding breach, unsupported labeling, or failure to follow escalation procedures.
Supported readiness	Safety thresholds are met, but targeted support is needed in one or more non-critical areas.	Safe routine responses with structured support for communication, documentation, or contextual reasoning.
Standard readiness	Competent performance across the five dimensions; routine supervision is appropriate.	Descriptive observation, proportionate response, sensitive communication, responsible documentation, and timely help-seeking.
Advanced readiness	Integrated and context-sensitive judgment across complex or ambiguous cases.	Balances cues, context, family perspectives, uncertainty, boundaries, and consultation, and revises decisions when needed.

These categories are provisional candidates for stakeholder co-design, formal standard setting, and empirical validation. They are not current practicum-entry standards or a basis for consequential decisions.

These provisional categories are intended to support differentiated preparation and supervision rather than simple labels of success or failure. In a future validated implementation, students classified as not yet ready would receive targeted remediation and reassessment before placement. Supported readiness would permit entry with an explicit supervision plan for specified non-critical needs. Standard and advanced readiness would indicate progressively more consistent and integrated performance, while still requiring normal practicum supervision.

Equity monitoring remains a domain-specific evaluation requirement. Institutions examine whether language, culture, disability, digital familiarity, or access conditions systematically affect ratings or recommendations and revise the assessment when such differences are not construct-relevant.

### 4.5 Domain 5: partnership governance

Partnership governance is the organizational and ethical infrastructure for the other four domains. Schools need practice-based knowledge of childcare routines, risks, and supervision capacity; childcare sites need agreed preparation standards, responsibilities, and escalation procedures before receiving students.

Responsibilities are distributed clearly. Educational institutions may take primary responsibility for curriculum design, student preparation, assessment documentation, and digital literacy. Childcare institutions may contribute practice-based standards, authentic scenarios, supervision expectations, and feedback regarding students’ transition into practicum. Mental-health, child-development, safeguarding, legal, and data-protection specialists may review higher-risk components. Technology providers remain accountable for system documentation, security, limitations, and changes in model behavior.

Governance also determines how implementation is evaluated. Adoption is not judged by platform usage, the number of generated scenarios, or the amount of student interaction alone. Relevant outcomes include acceptability to students and mentors, appropriateness for childcare contexts, feasibility within curriculum and supervision time, fidelity to agreed protocols, equity across student groups, educational effectiveness, and sustainability after pilot funding ends.

Evidence from implementation revises competency maps, scenarios, feedback protocols, readiness thresholds, and governance arrangements, sustaining the iterative cycle shown in **Figure 1**.

### 4.6 Operationalization and evaluation matrix

**Table 4** translates each domain into an educational purpose, bounded GenAI functions, retained human authority, and measurable indicators. Developmental-fidelity criteria draw on infant-specific pose modeling, real–synthetic validation, virtual infant development, and interactive digital-child research (17–20). Risks and safeguards are consolidated in Section 6 rather than repeated in the matrix.

**Table 4:** Operationalization and evaluation matrix for the five-domain framework.

Domain	Main purpose	Bounded GenAI functions	Retained human authority	Example evaluation indicators
Competency mapping	Define expected pre-practicum competence.	Draft or compare competency statements, rubrics, scenario mappings, and behavioral anchors.	Educators, childcare mentors, and relevant specialists approve dimensions, indicators, critical-error rules, and thresholds.	Expert consensus; clarity and relevance; curriculum and practicum alignment; inter-rater agreement.
Multimodal simulation	Provide controlled practice with sensitive or unevenly available situations.	Generate synthetic text cases, branching dialogue, staged media tasks, environmental observation activities, and validated child-like materials.	Professionals define age, cue, purpose, reference materials, acceptance thresholds, permitted use, and revalidation decisions.	Content validity; developmental plausibility; inter-rater agreement; error severity; learner misinterpretation; cultural review.
AI-supported reflective feedback	Convert performance into reflection and revision.	Summarize responses, compare with rubrics, generate prompts, suggest wording, and organize progress patterns.	Supervisors review, contextualize, revise, or reject feedback and resolve ambiguity.	Feedback accuracy; mentor revision or rejection rate; reflection quality; improvement across tasks; ability to critique AI output.
Readiness assessment	Support future practicum-entry decisions and identify support or remediation needs after satisfactory validation.	Organize portfolios, synthesize evidence, track progress, and flag items for human review.	Educators and childcare mentors make decisions, explain evidence, provide remediation, and manage appeals.	Threshold attainment; readiness distribution; remediation outcomes; assessor agreement; appeals; equity across groups.
Partnership governance	Maintain shared accountability and sustainable implementation.	Support audit records, data-use templates, version tracking, documentation, and incident workflows.	Partner institutions allocate responsibility, review incidents, oversee vendors, and approve changes or retirement.	Acceptability; appropriateness; feasibility; fidelity; equity; sustainability; protocol compliance; incident response.

Across all domains, GenAI performs bounded support functions. Educators, childcare mentors, and relevant professionals retain authority over standards, scenario approval, feedback interpretation, readiness decisions, and governance. Indicators are adapted to local curriculum and partnership capacity rather than treated as a universal checklist.

### 4.7 Testable research questions and candidate study designs

A rigorous program prespecifies the construct, assessment-development steps, comparators, measures, timing, and failure criteria. The following questions are sequential: measurement validation precedes comparative effectiveness, and predictive validity is examined subsequently.

RQ1 (instrument development, measurement validity, and generalization): Can the candidate PP-MHSCA blueprint be developed into a reliable, valid, accessible, and equitable assessment across raters, scenarios, modalities, and learner groups, and do students demonstrate competence in unfamiliar cases rather than reproduce model wording? A staged program uses expert content review, cognitive interviews, accessibility and scenario review, pilot item and station analysis, two blinded raters, novel transfer stations, generalizability analysis, transparent standard setting, and subgroup fairness checks. The assessment requires revision if evidence shows inadequate content representation, weak inter-rater agreement, unstable performance across stations, linguistic mimicry, construct-irrelevant access barriers, or meaningful differential item or scenario functioning.

RQ2 (comparative effectiveness after satisfactory measurement validation): Does human-supervised multimodal GenAI simulation improve validated PP-MHSCA outcomes, transfer to novel cases, and four- to six-week retention compared with (a) matched conventional simulation using the same competency targets, practice time, and human feedback but scripted or non-generative delivery, and (b) lecture plus case-discussion preparation? A randomized or cluster-randomized study would estimate group-by-time effects using blinded human ratings. Incremental value over conventional simulation requires superiority on at least one prespecified GenAI-specific endpoint, such as adaptive transfer, response flexibility, educator preparation time, or cost-effectiveness, without inferior safety, fairness, or core learning outcomes.

RQ3 (subsequent predictive validity): After the assessment has satisfactory measurement properties, do pre-practicum PP-MHSCA results predict independently assessed practicum performance? A prospective cohort obtains mentor and trained-observer ratings after four to eight weeks, using a standardized practicum rubric and, where appropriate, CLASS Infant/Toddler or Q-CCIIT/QCIT observations (45–47), together with documented supervision needs, family communication and documentation quality, and safety-critical incidents. Prediction is tested after adjustment for prior experience, academic performance, language background, and placement quality, with out-of-sample validation and calibration. Predictive value requires incremental information beyond baseline factors without subgroup miscalibration.

These questions make the framework testable; claims of effectiveness, validity, or prediction await the proposed studies. This ordering prevents developmental PP-MHSCA scores from being used as effectiveness outcomes or readiness evidence before acceptable measurement and fairness evidence is established.

## 5 Implementation pathway

**Figure 2:**
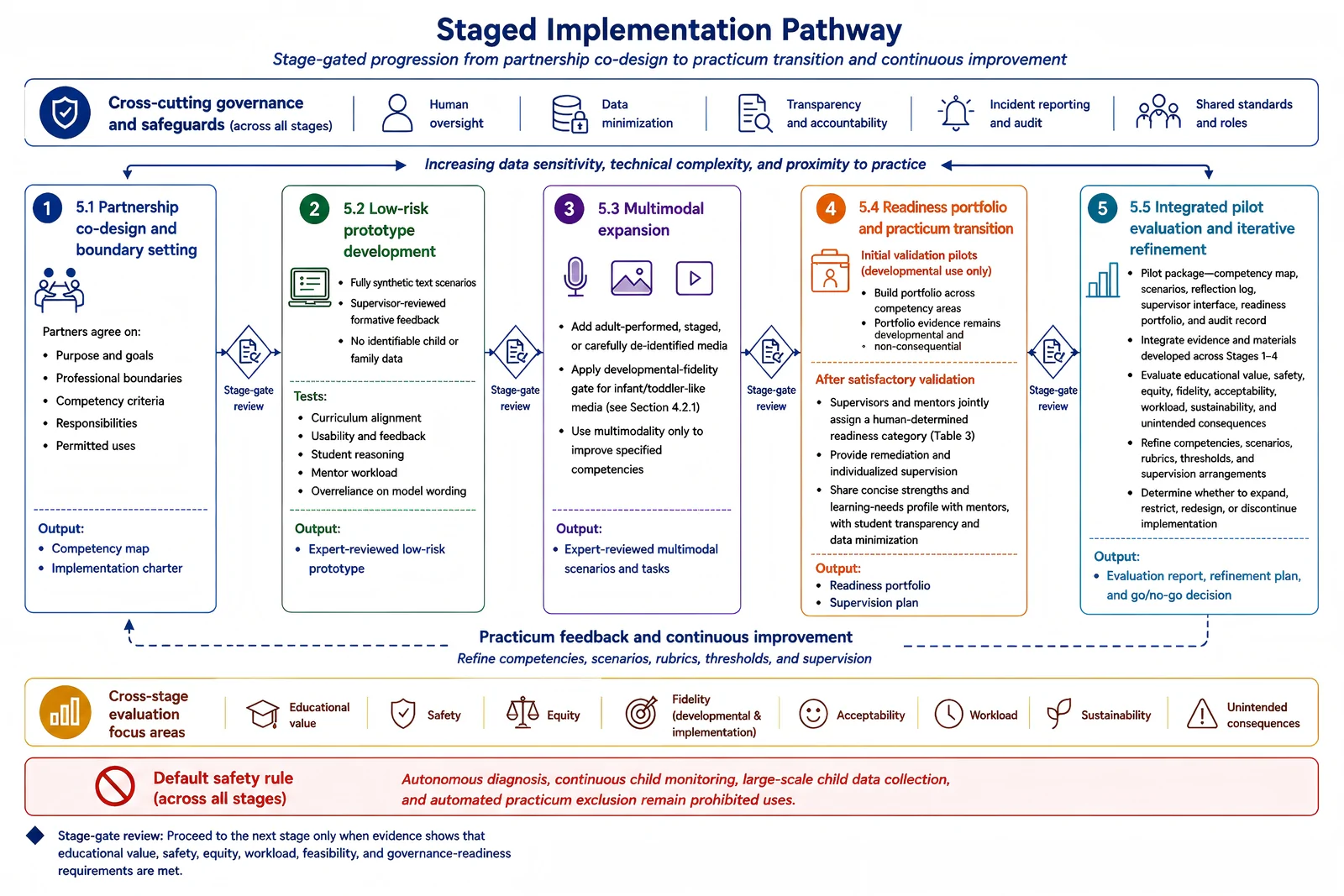
Staged implementation pathway for multimodal GenAI-supported pre-practicum mental health support competency development. The pathway progresses from partnership co-design and boundary setting through low-risk text-based prototype development and governed multimodal expansion to readiness portfolio development, practicum transition, and integrated pilot evaluation and iterative refinement. Progression between stages is contingent on stage-gate evidence of educational value, safety, equity, workload, feasibility, and governance readiness. The stages and gate criteria are proposed implementation hypotheses rather than validated implementation requirements. Cross-cutting governance, safeguards, and implementation evaluation apply throughout, including human oversight, data minimization, transparency and accountability, incident reporting and audit, shared standards and roles, developmental and implementation fidelity, acceptability, sustainability, and unintended consequences. Practicum feedback informs the continuous revision of competencies, scenarios, feedback processes, readiness thresholds, and supervision arrangements. Autonomous diagnosis, continuous child monitoring, large-scale child data collection, and automated practicum exclusion remain prohibited across all stages.

Implementation progresses from partnership co-design and low-risk prototypes to governed multimodal use, readiness portfolios, and supervised placement (**Figure 2**). Advancement depends on evidence of educational value, safety, equity, workload, and feasibility before data sensitivity, technical complexity, or proximity to practice increases.

### 5.1 Partnership co-design and boundary setting

Co-design precedes procurement. Education and childcare partners, students, family representatives, and relevant child-development, mental-health, safeguarding, and data-governance specialists agree on educational purpose, professional boundaries, competency criteria, responsibilities, and a limited set of permitted uses. The initial products are a competency map and implementation charter, not an AI platform.

### 5.2 Low-risk prototype development

The initial prototype uses fully synthetic text scenarios and supervisor-reviewed formative feedback without identifiable child or family data. It examines curriculum alignment, usability, feedback quality, student reasoning, mentor workload, and possible overreliance on model wording. Progression depends on evidence that students better distinguish observation from interpretation, consider alternatives, communicate sensitively, identify safety concerns, and seek supervision without unacceptable safety, equity, or workload effects.

### 5.3 Multimodal expansion

After review of the text prototype, partnerships may add adult-performed, staged, or carefully de-identified audio, video, or environmental images. Synthetic infant- or toddler-like media require the developmental-fidelity gate in Section 4.2.1. Multimodality is justified only when it improves a specified competency, such as interaction observation, attention to environmental influences, response to tone, or family communication. Students are taught that short clips provide incomplete evidence and are asked to state what is observable, what cannot be inferred, what context is missing, and when consultation is appropriate.

### 5.4 Readiness portfolio and practicum transition

Repeated simulations, reflective tasks, and supervisor-reviewed assessments form a readiness portfolio covering safeguarding, privacy, observation, responsive interaction, family communication, documentation, referral judgment, and critical AI literacy. GenAI may organize evidence and flag areas for review. Only after satisfactory assessment validation may educators and childcare mentors jointly assign a **Table 3** category; during initial pilots, portfolio evidence remains developmental and non-consequential. In a future validated implementation, a not-yet-ready category would trigger remediation and reassessment, whereas supported readiness would trigger an individualized supervision plan. At transition, mentors may receive a concise profile of strengths and learning needs, with student transparency and data minimization. Practicum feedback then refines competencies, scenarios, rubrics, and thresholds without creating continuous AI surveillance.

### 5.5 Integrated pilot evaluation and iterative refinement

Across the staged pathway, the initial integrated pilot remains deliberately modest: an expert-reviewed provisional competency map, 10–20 synthetic or staged scenarios, a reflection log, a supervisor interface, a readiness portfolio, and an audit record showing which AI suggestions were accepted, revised, or rejected. Autonomous scoring, live monitoring, large-scale child data, and predictive analytics are unnecessary at this stage. Evaluation integrates educational effectiveness with safety, equity, fidelity, acceptability, workload, sustainability, and unintended consequences. Evidence includes blinded competency ratings, mentor revision rates, incident and audit analysis, time and resource logs, accessibility and subgroup review where adequately powered, and qualitative feedback from students, educators, mentors, and relevant families. Usage and satisfaction are supplementary; null or negative results can halt progression. Proctor outcomes and RE-AIM can structure outcomes, while CFIR can interpret contextual determinants (12–14).

## 6 Ethical and governance safeguards

These safeguards apply across all domains and stages. They address data protection, human oversight, student rights, fairness, developmental fidelity, lifecycle controls, and organizational accountability. They draw on WHO AI ethics principles and proportionately adapt UNICEF’s child-centred AI requirements to a framework serving adult trainees rather than direct child-facing AI (7, 8, 49). **Table 5** is an illustrative crosswalk, not a claim of legal or regulatory compliance.

**Table 5:** Illustrative mapping to WHO AI ethics principles and UNICEF child-centred AI requirements.

Principle or requirement	Corresponding framework provisions
WHO AI ethics principles	
Protecting human autonomy	Human authority over consequential judgments; disclosure, correction, remediation, and appeal (6.1–6.2).
Promoting human well-being and safety and the public interest	No autonomous diagnosis or child-state inference; developmental-fidelity gate; cybersecurity and incident response (4.2, 6.2–6.3).
Ensuring transparency, explainability and intelligibility	Disclosure of AI use and limitations; version-traceable outputs, documentation, and audit trails (6.1, 6.3).
Fostering responsibility and accountability	Defined AI–human roles; named governance leads; vendor accountability; audit and incident procedures (6.2–6.3).
Ensuring inclusiveness and equity	Accessibility, cultural-validity, and subgroup review across language, disability, socioeconomic background, and digital access (5.5, 6.2).
Promoting AI that is responsive and sustainable	Lifecycle monitoring, revalidation, retirement, workload and resource review, and opportunity-cost assessment (5.5, 6.3, Section 7).
UNICEF child-centred AI requirements	
1. Ensure regulatory frameworks, oversight and compliance for child-centred AI	Child-rights review, joint governance, named roles, audit, redress, and local legal-policy review (6.3).
2. Ensure safety for children	Preference for synthetic or staged materials; prohibited autonomous inference; developmental and technical safety gates (4.2, 6.1–6.3).
3. Protect children’s data and privacy	Data minimization, modality-specific authorization, biometric and re-identification controls, retention limits, deletion, and vendor-use restrictions (6.1).
4. Ensure non-discrimination and fairness for children	Predeployment and ongoing fairness, accessibility, cultural-validity, and developmental-fidelity review with corrective thresholds (6.2).
5. Provide transparency, explainability and accountability for children	Plain disclosure, contestability, correction, remediation, appeal, audit trails, and incident response (6.1, 6.3).
6. Respect human and child rights through responsible AI practice	Bounded permitted uses, retained human authority, lifecycle governance, vendor obligations, and retirement criteria (6.2–6.3).
7. Support children’s best interests, development and well-being	Educational-necessity tests, developmental-fidelity validation, and safety-critical rules (3.5.1, 4.2, 6.1–6.2).
8. Ensure inclusion of and for children	Accessibility and digital-access review; proportionate family and child-rights representation in governance (6.2–6.3).
9. Prepare and empower children for present and future developments in AI	The framework trains adult students in critical AI literacy; any child-facing activity requires separate age-appropriate governance (3.5.1, 4.3).
10. Create an enabling environment for child-centred AI	Staged partnership implementation, multidisciplinary governance, capacity building, sustainable resourcing, and evidence sharing (Section 5, 6.3).

### 6.1 Data protection, transparency, and rights

Data use follows necessity, proportionality, minimization, purpose limitation, and child-centred protection. Synthetic, fictionalized, adult-performed, staged, or effectively de-identified materials are preferred when they meet the learning objective. Real child- or family-related data require documented educational necessity, an appropriate lawful basis, institutional approval, restricted access, defined retention, secure storage, and verifiable deletion. Identifiable information is not entered into unapproved general-purpose AI systems.

Video, audio, facial imagery, voice, and other potentially identifiable or biometric data receive heightened protection. Authorization specifies modality, purpose, storage, access, retention, and deletion; technical controls include encryption and access logging. Extraction of biometric identifiers, facial or voice embeddings, or emotion-recognition features requires separate lawful justification and independent review. A re-identification and child-rights risk assessment precedes use of de-identified audiovisual material. Child-related recordings are not used for model training, fine-tuning, or vendor product improvement without separate authorization and institutional review.

Students receive clear information about AI use, data collection, access, limitations, and any influence on learning or assessment. Readiness and progression decisions are explainable, contestable, and subject to human review. Students can inspect relevant evidence, correct inaccuracies, receive remediation, and appeal without retaliation. Formative use remains non-punitive, with reasonable alternatives for legitimate privacy, accessibility, or ethical concerns.

### 6.2 Human oversight, fairness, and developmental fidelity

AI is not used to diagnose, label, or infer a child’s emotional or mental state from behavioral, visual, vocal, or contextual signals. Nor does it autonomously certify competence, determine readiness, exclude students from practicum, or make other high-stakes educational decisions. It may support scenario generation, evidence organization, formative feedback, and reflection, while qualified educators and childcare mentors retain final authority.

Consequential outputs are reviewed against multiple evidence sources. Readiness, progression, and remediation decisions are documented; lower-risk formative outputs may be reviewed through proportionate sampling. Institutional protocols define AI–human responsibilities and procedures for escalating uncertainty, disagreement, errors, or harmful outputs (7, 8).

Bias, accessibility, cultural validity, and developmental fidelity are assessed before deployment and monitored during use. Review may include accessibility testing, cognitive interviews, structured subgroup review, subgroup comparisons, and, when psychometrically appropriate, differential item functioning. Methods are proportionate to instrument maturity, sample size, data sensitivity, and deployment risk.

Findings and corrective actions are documented. Construct-irrelevant disparities, recurring harms, serious accessibility barriers, or persistent developmental errors trigger revision, restriction, suspension, or retirement. Synthetic or staged child representations remain subject to the developmental-fidelity gate in Section 4.2.1, version control, and revalidation after material change.

### 6.3 Lifecycle, technical, and organizational governance

Governance covers design, validation, deployment, monitoring, updating, and retirement. Core documentation records educational purpose, intended users, permitted and prohibited functions, data flows, child-rights implications, foreseeable risks, and non-AI alternatives. Validation addresses content, developmental fidelity, safety, fairness, accessibility, usability, and technical readiness; deployment follows Section 5.

Monitoring covers educational value, safety and privacy incidents, equity, feedback quality, workload, resource use, and audit completeness. Material changes to models, prompts, scenarios, rubrics, data pipelines, interfaces, or intended uses undergo proportionate revalidation and controlled change management. Version records, approval responsibilities, testing, rollback, user notification, and retirement arrangements are documented.

Technical controls are proportionate to risk and data sensitivity and may include role-based access, strong authentication, encryption, secure configuration, vulnerability testing, backup, rollback, and incident response. Governance also addresses data leakage, prompt injection, insecure integrations, malicious inputs, and misuse of exported content. A validated benchmark is rerun after material updates and at defined intervals; threshold breaches trigger revalidation, restriction, rollback, or suspension.

A joint governance body includes education and childcare leaders, educators or mentors, student representatives, and relevant family, child-rights, safeguarding, privacy, technical, or developmental expertise. A named safeguarding lead may pause activities that raise child-protection concerns; a privacy lead oversees authorization and data handling; and a system owner is accountable for validation, monitoring, and change control.

Vendor agreements cover documentation, data handling, subprocessors, security, limitations, material-change and breach notification, audit cooperation, and secure exit. Audit trails record system versions, authorized input provenance, consequential outputs, and human decisions. Incident procedures specify reportable events, severity, responsibilities, containment, notification, remediation, and escalation. Governance is reviewed periodically and after material system or regulatory change.

WHO and UNICEF principles are normative reference points, not substitutes for applicable law or institutional policy. Because the framework serves adult trainees, child safety, privacy, fairness, best interests, development, and rights apply directly; children’s participation and AI literacy are only partly applicable unless direct child-facing components are introduced.

## 7 Discussion

As a conceptual analysis, this study was designed to generate an evidence-informed synthesis and a testable framework. The outputs should therefore be interpreted at three levels.

First, the framework’s foundational principles are evidence-informed. These include responsive caregiving, safeguarding, clearly defined role boundaries, human oversight, simulation-based learning, reflective feedback, and the use of multiple sources of assessment evidence. They were derived from relevant professional standards, normative guidance, and the direct-component, adjacent, analogical, and normative sources described in **Supplementary Appendix S1**.

Second, the integration of these elements into a five-domain model is a conceptually derived and evidence-informed architecture produced through the present analysis. The configuration of the domains, their proposed relationships, and the staged pathway constitute the principal conceptual contribution, but the complete architecture has not yet been empirically evaluated.

Third, the four-level competency model, readiness categories, critical-error definitions and override rules, scoring and aggregation procedures, cut scores and thresholds, **Table 2** acceptance rules, and PP-MHSCA station formats are presented as structured hypotheses and candidate specifications for stakeholder co-design, professional and safeguarding review, pilot testing, formal standard setting, accessibility and fairness evaluation, and empirical validation before implementation or use in consequential practicum-readiness decisions.

This paper positions multimodal GenAI as a supervised pre-practicum extension of established simulation, not as a substitute for authentic practicum or professional judgment. This placement is deliberately cautious: current mental health-related AI remains limited in validity, safety, bias control, generalizability, and deployment readiness (4, 5, 37–39), whereas technology-enhanced simulation evidence supports the educational value of structured practice, feedback, repetition, and assessment (42). In a protected learning environment, inaccurate or incomplete outputs can be examined and corrected before they affect children or families.

The proposed incremental value is conditional rather than universal. GenAI may add adaptive scenario variation, contingent dialogue, multimodal cue integration, and individualized formative prompts at scale (30–32), but conventional role-play, validated video cases, standardized encounters, and human facilitation may provide greater stability, relational fidelity, and control. GenAI is most defensible when these additional affordances address a defined learning problem and their anticipated educational value outweighs the costs of validation, infrastructure, staff preparation, monitoring, and revalidation.

A central counterargument is that AI investment may divert resources from low pay, turnover, staffing shortages, mentor capacity, and practicum support. An additionality and opportunity-cost test is therefore necessary: AI spending should not displace staffing, supervision, mentor release time, or student support; full costs include expert review, workload, accessibility, maintenance, and vendor dependence; and continuation requires benefits that credible lower-technology alternatives cannot provide. Otherwise, workforce and mentoring investment takes priority.

A second counterargument is that AI may increase rather than reduce workload and may narrow professional learning by encouraging formulaic responses, surveillance, or automation bias. Educators may spend substantial time validating scenarios and correcting feedback, while students may learn to imitate model language rather than exercise judgment. Evaluation therefore includes mentor revision and rejection rates, educator time, student dependence, transfer to novel cases, unsafe or overconfident responses, accessibility, subgroup effects, and unintended consequences—not only satisfaction or usage. The testable pathway in Section 4.7 allows null or negative findings to count against adoption.

Validation needs to be considered at two levels. Framework-level evaluation should examine the relevance, completeness, comprehensibility, contextual appropriateness, feasibility, and relationships of the five proposed domains through co-design with trainees, educators, practicum supervisors, childcare providers, safeguarding leads, families or family representatives where appropriate, and relevant professional or regulatory stakeholders. Assessment-level validation should subsequently examine the PP-MHSCA blueprint, station sampling, scoring rubrics, competency levels, readiness categories, critical-error rules, cut scores, classification consistency, accessibility, fairness, and decision consequences. Until both levels of evidence are adequate for the intended use, the framework should support research, curriculum deliberation, and supervised pilot development rather than consequential practicum-entry decisions.

The framework should therefore be understood as conceptually transferable but operationally context-dependent. Its core architecture is based on broadly applicable professional and governance principles, while China is proposed as the initial context for implementation and empirical evaluation. Chinese national requirements inform the first implementation pathway rather than defining the framework’s universal content. Use in another jurisdiction would require local co-design, substitution of applicable professional, safeguarding, data-protection, and practicum requirements, and renewed evaluation of contextual fit, feasibility, fairness, and validity.

The evidence base remains indirect. Research from health-professions simulation, teacher education, and digital-child projects cannot establish effectiveness in pre-service childcare education. Claims about developmental fidelity require local expert validation, and readiness claims require prospective evidence that pre-practicum scores predict placement performance. Culture, legal requirements, institutional capacity, and mentor availability will shape feasibility. GenAI-supported preparation is therefore a bounded complement to—not a remedy for—investment in the childcare workforce and practicum system.

## 8 Conclusion

Multimodal GenAI may offer a useful, supervised bridge between campus-based preparation and authentic childcare practicum, particularly where students otherwise have limited opportunities to rehearse sensitive, ambiguous, or ethically difficult situations. Its educational contribution remains conditional on developmental fidelity, human oversight, proportionate data governance, equitable access, and evidence of added value over credible conventional alternatives.

The proposed framework integrates competency mapping, multimodal simulation, AI-supported reflective feedback, readiness assessment, and partnership governance. It is evidence-informed in its foundations, conceptually derived in its integrated architecture, and empirically testable in its operational specifications. Future research can determine whether these arrangements improve competency development and practicum transition without displacing investment in educators, mentors, safeguarding, or the wider childcare workforce. Implementation nevertheless requires context-specific adaptation and validation.
