# Supplementary material for: Multimodal generative AI for pre-practicum mental health support competency development: a human-supervised framework for education–childcare partnerships
Source: Front Psychiatry. 2026 Aug 31;17:1922251. doi: 10.3389/fpsyt.2026.1922251 (PMC13572418; doi:10.3389/fpsyt.2026.1922251)
Supplement: Supplementary file 1 [file SupplementaryFile1.docx]

SUPPLEMENTARY MATERIAL

Supplementary Appendix S1

Literature Identification, Concept Extraction, and Synthesis Procedures

# S1.1 Methodological orientation

This study used conceptual analysis to clarify the meaning, dimensions, professional boundaries, and educational implications of pre-practicum mental health support competence. The purpose was not to conduct an exhaustive systematic review or estimate the effects of a specific intervention. Instead, the literature was used to identify relevant concepts, compare their meanings and relationships, clarify boundary conditions, and develop an integrated conceptual framework.

The analytical procedure was informed by Petrina’s account of conceptual analysis (28), particularly the processes of identifying a conceptual problem, resolving semantic variation, mapping conceptual dimensions, establishing referential boundaries, and developing a revised conceptual representation.

# S1.2 Literature identification

Literature searches were conducted in PubMed, Scopus, Web of Science, ERIC, and Google Scholar. Backward and forward citation searching was also used to identify relevant sources cited by, or subsequently citing, key publications. Targeted searches were conducted for authoritative guidance and policy documents issued by organizations such as the World Health Organization, UNICEF, the National Institute of Standards and Technology, professional bodies, and relevant Chinese regulatory authorities.

Sources published or available through 5 August 2026 were considered. This literature cut-off reflects the date on which the evidence base for the revised manuscript was finalized.

Searches combined terms from the following concept groups:

- generative artificial intelligence, large language models, large multimodal models, and conversational agents;
- mental health, mental health support, and psychosocial support;
- childcare, infant and toddler care, early childhood education, and early childhood mental health;
- responsive caregiving, observation, family communication, safeguarding, referral, and documentation;
- simulation, virtual patients, simulated patients, standardized actors, mixed reality, video observation, and conversational practice;
- competency-based education, competency assessment, work-integrated learning, and practicum readiness;
- implementation science, governance, child rights, privacy, bias, equity, cybersecurity, accountability, and model monitoring.

Search terms were combined and adapted according to the terminology and indexing conventions of each database. The searches were intended to support conceptual coverage and boundary clarification rather than exhaustive retrieval.

# S1.3 Literature streams and source selection

The analysis drew primarily on five literature streams:

1. GenAI and large language models in mental health;
2. multimodal simulation and conversational practice;
3. responsive caregiving and early childhood mental health;
4. competency-based education and work-integrated learning; and
5. AI governance and implementation science.

Cross-domain evidence was also incorporated from teacher-education simulation (29), GenAI-supported virtual- and simulated-patient methodologies (30–32), and infant and early childhood mental health competency and consultation frameworks (33–36).

Sources were selected purposively according to four considerations:

1. theoretical relevance to the proposed competency framework;
2. contribution to the definition or differentiation of a concept;
3. applicability to childcare education and pre-practicum preparation; and
4. capacity to clarify professional, ethical, developmental, or technological boundaries.

Priority was given to peer-reviewed reviews, directly relevant empirical studies, established competency or professional frameworks, and primary governance and policy documents. A source was retained when it contributed at least one necessary concept, pedagogical mechanism, professional boundary, implementation condition, assessment consideration, or governance requirement.

Sources were generally excluded when they focused exclusively on autonomous diagnosis or treatment, had no clear relevance to education or supervised preparation, addressed technical performance without educational or governance implications, or duplicated a conceptual contribution already represented by a more authoritative source. The proximity, intended analytical function, and transferability of evidence from different professional fields are described in Section S1.4.

# S1.4 Evidence proximity and transferability

No substantive source directly evaluated the complete proposed intervention in the target population and practice context. Given this limitation, each substantive source was assigned to the category that best reflected both its proximity to the target context and its primary function within the conceptual analysis. These categories describe contextual proximity and the permissible scope of evidence transfer; they do not represent a hierarchy of methodological quality. Reference 28 informed the conceptual-analysis methodology and was therefore considered separately from the substantive evidence-proximity categories.

Direct component sources were located within one or more elements of the target population, age range, professional role, practice setting, or observable content addressed by a component of the framework. These sources comprised responsive-caregiving guidance, early-childhood professional standards or infant–toddler educator competencies, and measures of infant–toddler caregiver–child interaction (2,3,34,45–47). References 2, 3, and 34 informed the identification of observable caregiving practices and educator responsibilities, whereas references 45–47 informed complementary indicators of caregiver–child interaction quality. These sources supported bounded content decisions.

Adjacent sources addressed a closely related developmental, educational, mental-health, professional, or assessment context, but not the precise intersection of pre-service childcare education, infant–toddler mental-health support, and GenAI-supported preparation. This category included the nurturing-care framework; research on the contextual interpretation of child emotion; preschool behavioral screening; work-integrated-learning equity; infant and early childhood mental-health competency and consultation frameworks; conceptual and empirical literature on GenAI in early childhood education; AI literacy; and graded competency assessment (1,9–11,15,16,33,35,36,40,41,43,44). These sources were used cautiously to clarify construct boundaries, developmental considerations, educational conditions, and provisional assessment principles.

Analogical sources came from mental-health AI and conversational-agent research, largely involving adult or mixed-age populations; implementation-science frameworks; teacher education; health-professions training using virtual or simulated patients; infant pose-estimation and digital infant or child modeling and simulation; and automation research (4–6,12–14,17–20,29–32,37–39,42,48). In the absence of directly matched intervention studies, these sources contributed to the conceptual synthesis by identifying possible mechanisms, implementation constructs, comparators, technical failure modes, and questions requiring empirical investigation. However, because learner roles, developmental characteristics, safeguarding responsibilities, relational continuity, organizational settings, and practice conditions differ, they were not assumed to be directly transferable or to demonstrate feasibility, effectiveness, construct validity, predictive validity, or generalizability in the target context.

Normative and reporting sources consisted of WHO and UNICEF guidance, AI-intervention reporting guidance, and applicable Chinese legal, regulatory, childcare, safeguarding, and practicum policy sources (7,8,21–27,49–51). These documents informed governance principles, reporting expectations, child-rights protections, data handling, accountability, and requirements for local legal review. They provide normative, reporting, or regulatory direction rather than empirical evidence of educational effectiveness.

Accordingly, direct component sources were used to support bounded content claims; adjacent and analogical sources were used for conceptual integration and to generate provisional construct, design, implementation, and evaluation propositions; and normative and reporting sources specified governance, reporting, and legal constraints. This use of indirect evidence is intended to support the development of a conceptually grounded framework and a set of testable propositions, rather than to substitute for evidence generated in the target context. The developmental fidelity, feasibility, safety, equity, measurement properties, educational effectiveness, and predictive value of the integrated framework therefore remain to be established through contextual co-design and empirical testing with pre-service childcare professionals preparing to work with infants and toddlers.

# S1.5 Concept extraction

Relevant information was extracted from each source according to its conceptual contribution. The extraction focused on:

- definitions and uses of key concepts;
- defining and recurring attributes;
- pedagogical mechanisms;
- observable competency indicators;
- professional roles and limits;
- developmental and safeguarding considerations;
- assessment and readiness criteria;
- implementation conditions;
- governance and accountability requirements; and
- reported risks, tensions, or unintended consequences.

The analysis distinguished between defining attributes and features that were only contextually associated with the concept. Related and contrasting concepts were compared to clarify the distinction between educational mental health support and clinical diagnosis or treatment, and between AI-supported learning and autonomous AI assessment or decision-making.

# S1.6 Conceptual synthesis

The extracted concepts were compared across the five literature streams to identify areas of convergence, conceptual tension, and complementarity. Candidate elements were then mapped onto the pre-practicum educational workflow.

Elements were retained as separate domains when they represented a conceptually distinct and educationally necessary function. Overlapping elements were combined, whereas elements falling outside the non-clinical and pre-practicum scope were excluded or treated as boundary conditions.

The provisional domains were examined against relevant counterexamples and alternative cases, including:

- conventional simulation without GenAI;
- synthetic child cues that were developmentally inaccurate or misleading;
- institutions with limited technical or supervisory resources;
- the use of AI-generated scores in high-stakes assessment; and
- applications that crossed from educational support into diagnosis, surveillance, or autonomous decision-making.

This iterative process resulted in five interconnected design domains: competency mapping, multimodal simulation, AI-supported reflective feedback, readiness assessment, and partnership governance. The resulting framework was used to clarify shared characteristics, professional and ethical boundaries, implementation requirements, and testable research propositions.

# S1.7 Author roles and analytical review

The initial literature identification and concept extraction were conducted by Wenlan Xie and Chunye Xiang. The provisional concepts and domain structure were independently reviewed by Fan Wu. Xingda Chen provided supervisory review. Differences in interpretation, evidence classification, or domain placement were discussed among the authors until agreement was reached. All authors reviewed the final five-domain framework for conceptual coherence, relevance to pre-practicum education, and consistency with the non-clinical professional boundaries of the study.

# S1.8 Methodological scope and limitations

Because this study was a conceptual analysis rather than a systematic review, it did not use PRISMA-based study selection, formal risk-of-bias assessment, or quantitative evidence aggregation. The purposive selection of sources may not capture every relevant publication, and the proposed framework should therefore be regarded as provisional.

The framework provides a conceptually grounded structure for subsequent empirical investigation rather than a psychometrically validated competency construct. Future research should examine its content validity, structural coherence, feasibility, equity, safety, and predictive value across different educational and childcare contexts. It should be treated as a hypothesis-generating conceptual structure awaiting empirical co-design and testing.
